# Shifting Trend of Protein Consumption in Southeast Asia: Toward Health, Innovation, and Sustainability

**DOI:** 10.1016/j.cdnut.2024.104443

**Published:** 2024-08-18

**Authors:** Alvin Surya Tjahyo, Jia Yee Wu, Geoffry Smith, Cecilia Acuin, Andrea B Maier, Shaun Yong Jie Sim, Reshma Taneja, Sumanto Haldar, Christiani Jeyakumar Henry

**Affiliations:** 1 Clinical Nutrition Research Centre (CNRC), Singapore Institute of Food and Biotechnology Innovation (SIFBI), Agency for Science, Technology and Research (A∗STAR), Singapore, Singapore; 2 Essential Micronutrients Foundation, Singapore International Life Sciences Institute (ILSI), South East Asia Region, Singapore, Singapore; 3 Institute of Human Nutrition and Food, University of Philippines Los Baños, Los Baños, Philippines; 4 Centre for Healthy Longevity, @AgeSingapore, National University of Singapore, Singapore, Singapore; 5 Healthy Longevity Translational Research Program, Yong Loo Lin School of Medicine, National University of Singapore, Singapore, Singapore; 6 Department of Human Movement Sciences, @AgeAmsterdam, Vrije Universiteit Amsterdam, Amsterdam Movement Sciences, Amsterdam, the Netherlands; 7 Department of Physiology, Healthy Longevity Translational Research Program, Yong Loo Lin School of Medicine, National University of Singapore, Singapore, Singapore; 8 Faculty of Health and Social Sciences, Bournemouth University, Bournemouth, United Kingdom; 9 Department of Biochemistry, National University of Singapore, Singapore, Singapore

**Keywords:** dietary protein, protein intake, protein quality, complementary and alternative protein, future foods, Southeast Asia

## Abstract

Complementing discourse following a February 2023 event on dietary protein needs in Southeast Asia (SEA), this symposium report summarizes the region’s protein intake, while simultaneously examining the impact of dietary shift toward complementary and alternative proteins and their health implications. It highlights the importance of protein quality in dietary evaluations, optimal intake, and sustainability, advocating for environmentally conscious protein production and innovation in future foods. Discussion points, expert opinions, national nutrition data, and relevant literature, addressing protein intake and quality, their impact on human health, and various technologies for future foods production, have been included. Despite increased protein supply in SEA, protein requirements, particularly during crucial life stages, are often unmet owing to insufficient focus on protein quality. Factoring in amino acids content and bioaccessibility are crucial for assessing nutritional requirement and sustainability evaluations, rather than solely relying on protein quantity alone. Different food sources of protein also have different key conutrients for health relevance such as vitamin B-12 and ω-3 fatty acids. Innovations in food structure, processing, and technology are key to developing nutritious, sustainable, and appealing future foods, including from complementary and alternative protein sources, while considering safety aspects, especially allergenicity. Addressing protein needs in SEA requires a dual focus on protein quantity and quality, underlining the role of public health policies and guidelines that consider key nutritional differences of animal-source and plant-based proteins. To address regional demands, future food innovations should aim at creating unique yet needful food categories or supplementing current existing sources, rather than mimicking current products.

## Introduction

Proteins play a multifaceted role in human physiology, distinguishing them from carbohydrates and lipids, which are primarily used for energy production or storage. Dietary proteins not only serve as an energy source but also supply the amino acids as the fundamental building blocks for various bodily structures and functions. This underscore protein as a vital macronutrient for both the development and maintenance of health across the lifespan of humans, requiring an understanding of the optimal quantity and quality of its consumption.

Southeast Asia (SEA), a region consisting of Brunei, Cambodia, East Timor, Indonesia, Laos, Malaysia, Myanmar, Philippines, Singapore, Thailand, and Vietnam, is home to 8.4% of the world’s population [1] and some of the fastest growing economies [2]. As incomes increase, there has been a notable shift from predominantly plant-based to animal-based protein consumption [3,4]. Simultaneously, most Southeast Asian nations have witnessed an increase in per capita food availability, including proteins [5]. Both the increase in demand for and production of animal-based protein have raised concerns about environmental sustainability and the climate impact [6]. This article presents findings from the International Life Sciences Institute, SEA Region Symposium held in February 2023, centered on dietary protein needs in SEA, which aimed the following: *1*) to investigate protein intake in SEA, its health implications, and impact on the environment; and *2*) to discuss how food technology and processing can help address the protein needs of populations, with a focus on vulnerable groups.

## Summary Synthesis of Presentations

### Assessing protein availability and intakes in SEA

**FIGURE 1:**
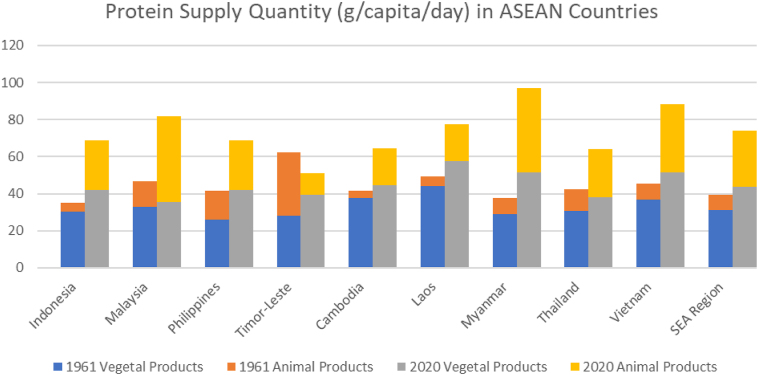
Comparison of protein supply quantity per capita (g/d) from the Food Balance Sheet from different countries within Southeast Asia between 1961 and 2020 [7]. The 2020 data from Brunei and the 1961 and 2020 data from Singapore are not available.

To assess the protein intake on a population level, food balance sheets provide macrolevel perspective of the available nutrients supply at a given point in time. Figure 1 reveals that both plant and animal protein availability has increased across the Southeast Asian region from 1961 to 2020 [7]. Notably, the increase in animal protein availability is also proportionally higher than the increase in plant protein availability across Southeast Asian countries, except for Timor-Leste. Professor Cecilia Acuin pointed out that these increases in protein availability, however, do not necessarily reflect actual protein consumption concentrations within the population, because protein availability statistics often include protein quantities produced for export purposes, which may not be accessible for local consumption.

Despite the reported increase in protein availability (Figure 1), protein intakes across the region do not always meet the recommended dietary allowance (RDA), particularly during life stages with high-metabolic demands, such as pregnancy, lactation, and childhood [8,9]. These demographics require elevated protein requirements to support various metabolic demands, such as fostering fetal growth, child development, and milk production in pregnant and lactating women.

**FIGURE 2:**
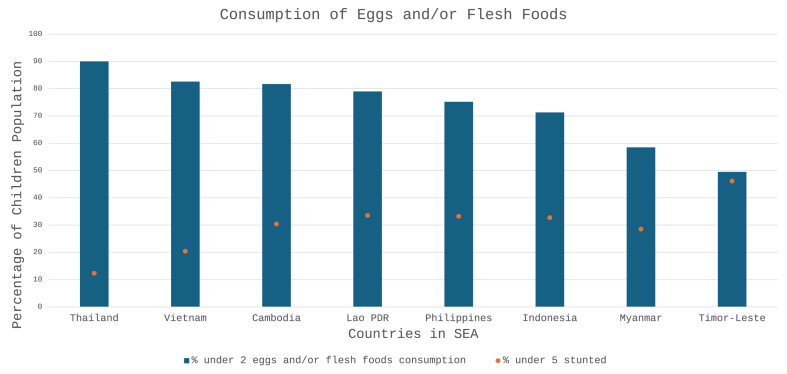
Comparative analysis of consumption of eggs and/or flesh foods and stunting in Southeast Asian countries. The blue bars represent the percentage of children aged <2 y in each country consuming eggs and/or flesh foods. The orange dot traces the percentage of children aged <5 y who are stunted.

Studies show that <30% of pregnant and lactating mothers population meet the estimated average requirement for protein [10,11]. Further exacerbating this issue, studies from Indonesia and Vietnam consistently reveal protein intake deficiency among children aged 0.5–11 y, leading to a high prevalence of undernutrition and potentially contributes to stunting especially in rural areas [12,13]. In SEA, a higher stunting rate is observed in children from countries with lower intake of animal-source protein-rich foods [14]. As shown in Figure 2, a negative association is observed between consumption of eggs and/or flesh foods in children aged <2 y with the percentage of stunting in children aged <5 y in different countries within the region [14,15]. Stunting is associated with a range of metabolic disorders, reduced cognitive function, and loss of productivity potential in adulthood [16]. Furthermore, there has been evidence linking total protein intake with better outcomes in growth trajectories [17,18], emphasizing the need to meet protein quantity requirements as early as possible in childhood development.

On the contrary, elderly populations, require specialized nutritional attention primarily to prevent muscle loss and atrophy. Because of greater anabolic resistance during aging, a higher protein intake is necessary in older individuals to maintain similar muscle mass as their younger counterparts [19].

To address these needs, it has been proposed that older adults aged ≥65 y require higher protein intake compared with normal healthy adults aged 18–64 y, at 1.0–1.2 g/kg of body weight/d (25%–50% increase from current RDA of 0.8 g/kg body weight/d for normal healthy adults) [20,21]. However, a significant portion of individuals within this demographic cohort fails to fulfill this increased protein demands [22], especially during catabolic crises, such as hospitalization and muscle loss [23,24].

Furthermore, meeting adequate protein quantity alone is often not enough. The critical importance of protein quality is often overlooked in favor of quantifying total protein intake, regardless of protein sources.

### Importance of protein quality in SEA

Protein quality, characterized by factors such as indispensable amino acid (IAA) pattern and protein digestibility, are important in addressing nutritional deficiencies. A linear programming model conducted in 10 Indonesian districts with high-stunting prevalence revealed adequate protein intake among the population, pointing to deficiencies in protein quality rather than quantity being a crucial factor in stunting prevention [25]. In Thailand, Kittisakmontri et al. [26] noted that animal-based protein positively correlated with weight gain and growth-related hormones in infants aged <1 y, a relationship not mirrored by plant-based proteins. Taken together, these findings highlight the differential impact of protein sources on growth and development, emphasizing the need for a nuanced understanding of protein quality in dietary recommendations.

Of course, the importance of protein quality brings focus on the metrics and various methods to measure it. Protein Digestibility Corrected Amino Acid Score (PDCAAS) [27] and Digestible Indispensable Amino Acid Score (DIAAS) [28] are the most common metrics for protein quality evaluation, with both measures being based on the IAA requirements of a reference population, with PDCAAS scored from total tract fecal protein digestibility and DIAAS from true ileal digestibility of each amino acids. DIAAS is a more accurate method, as measurement of ileal digestibility is more reflective of amino acids digestion and absorption, which occurs in the small intestine instead of the colon.

In vivo methods for tracking dietary protein’s fate after absorption into the body include the dual isotope tracer method and the indicator amino acid oxidation slope method. Dual isotope tracer method measures true amino acid digestibility and absorption kinetics but is limited by the complex requirement for intrinsic labeling, making it challenging and impractical [29]. On the contrary, the indicator amino acid oxidation slope measures the metabolic oxidation of ^13^C-labeled IAAs as surrogates for protein synthesis, hence bypassing the need for intrinsic labeling of test proteins. However, this method is limited to assessing each amino acid individually at a time and requires repeated measurements [29].

In vitro protein digestibility methods also offer a noninvasive approach to assess protein quality. These methods usually involve multienzyme system composed of digestive enzymes and manipulations of the buffer pH to mimic the gastrointestinal tracts. These in vitro digestibility methods, however, may not accurately reflect the complex interactions and digestive processes that occur in the human body, such as interference from antinutritional factors in foods and the enzymes secreted in response to different foods [30].

Although there are several methods available to measure protein digestibility, Associate Professor Wantanee Kriengsinyos stressed that the fundamental limitation is the lack of rigorous testing for agreement between the different methods and comparability between studies. This severely limits the ability to pool the different protein quality data to build a comprehensive database. Despite the limitations, the INFOGEST static in vitro digestion protocol is an ideal and robust tool to determine the DIAAS values of protein foods. This is due to its noninvasiveness, fast adaptation to different conditions, its potential for high reproducibility, and it being recently well-validated against in vivo data [31], thereby highlighting its potential utility as a harmonized tool for protein quality measurement.

### Maximizing health benefits with protein: nutritional evidence and public health recommendations

#### Optimizing animal-based compared with plant-based protein sources for health and sustainability

**FIGURE 3:**
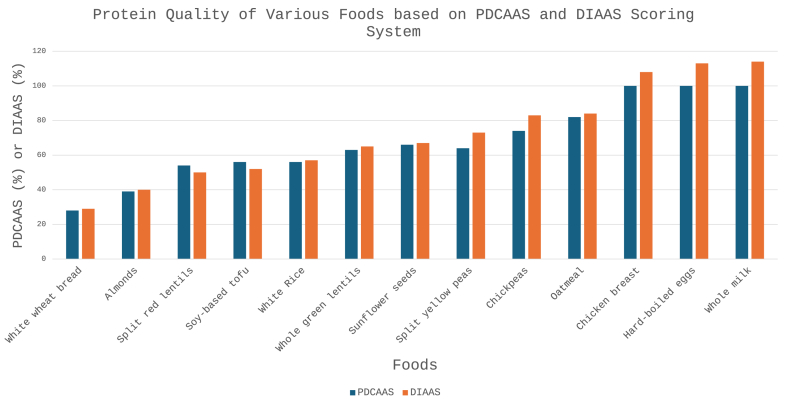
Comparison of protein quality in various foods evaluated using PDCAAS and DIAAS scoring systems. Each pair of bars represents the protein quality score of a particular food, with the blue bar indicating the PDCAAS score and the orange bar reflecting the DIAAS score. DIAAS, Digestible Indispensable Amino Acid Score; PDCAAS, Protein Digestibility Corrected Amino Acid Score.

PDCAAS and DIAAS scores, as shown in Figure 3, reveals notable variations in protein quality, particularly between proteins of animal and plant origins [32]. Although substituting diets high in animal-based foods with more plant-based foods has been linked to better health outcomes [33] and increased sustainability, it remains unclear whether plant-based proteins can fulfill the essential amino acid requirements as effectively as animal-based proteins. As shown in Figure 3, animal-based protein sources’ DIAAS scores are often >100 whereas most plant-based protein sources’ scores are <75 (except for chickpeas and oatmeal), indicating that the latter have less optimal amino acid distribution. This means that individuals consuming exclusively plant-based foods need to consume a wider variety of plant-based protein foods to complement each food’s deficiency in specific amino acids.

#### Protein quantity and quality for muscle health and maintenance

Older adults aged ≥65 y often require higher protein intake than younger individuals to maintain muscle mass [19,34]. Studies have shown that older adults with the highest quintile of protein intake lose ∼40% less total lean mass and appendicular lean mass compared with those with the lowest quintile of intake [35], emphasizing the need for an optimal RDA for muscle mass maintenance in this age group.

Sarcopenia characterized by diminished muscle mass, strength, and function [36,37] frequently remains undiagnosed and untreated [38] and pose a significant clinical burden. Professor Andrea Maier further stressed that knowledge and awareness regarding sarcopenia are lacking even among medical practitioners and underscored the necessity for further research, particularly regarding the optimal timing and protein quality needs for elderly individuals to effectively combat sarcopenia [39].

There exists conflicting research regarding the impact of animal and plant protein on human muscle growth and strength. Two meta-analyses concluded no differences in absolute lean mass and strength change between groups supplemented with animal or plant protein [40,41]. Lim et al. [40], however, did find a favoring effect of animal protein on absolute and percent lean mass in a subset of younger adults (<50 y).

Branched-chain amino acids such as leucine, isoleucine, and valine play key roles in muscle anabolism [42]. Prof Stuart Philips identified that a pivotal factor in maintaining cellular protein homeostasis is the mammalian target of rapamycin complex (mTORC) 1, activated by branched-chain amino acids. Leucine supplementation has demonstrated positive impacts on muscle protein synthesis [34]. More importantly, these anabolism signals must be bolstered by sufficient amino acids to sustain muscle protein synthesis. For instance, along with an increase in total protein intake, leucine supplementation has been observed to increase lean body mass in healthy adults [43] and increase muscle mass, strength, and physical functions in sarcopenic poststroke patients [44].

Although mTORC1 activity is essential for muscle growth, its sustained activation can disrupt protein homeostasis and contribute to muscle damage. This is because mTORC1 inhibits the subsequent autophagy process, which is responsible for removing damaged proteins [45]. Targeting the 4E-binding protein, downstream of mTORC1 involved in protein synthesis but not autophagy, restored protein homeostasis in mouse models of sarcopenia [46]. Assistant Professor Shih-Yin Tsai highlighted that this approach holds therapeutic potential for sarcopenia in the future.

#### Protein quantity and quality for optimal immune functions

Professor Philip Calder addressed the diverse role of amino acids on immune functions. In vitro*,* L-arginine is rapidly metabolized by activated T cells and regulates downstream metabolic pathways, crucial in proliferation, differentiation, and survival of human T cells [47]. In clinical practice, nutrition therapy involving arginine supplementation preoperative and postoperative has been associated with significant reduction in infection and shorter length of hospital stay [48]. Similarly, tryptophan is heavily involved in the immune response through the kynurenine pathway, involving both T and B cells [49].

Amino acids also serve as energy sources, metabolic regulators, substrates for biosynthesis, and they exert effect on the gut microbiota [50,51], all of which collectively shape immune system integrity. As plant foods tend to be lower in protein density than animal foods, it is important for individuals on exclusively plant foods diet to have a variety of plant sources of proteins in order to ensure sufficient protein intake.

#### Protein quantity and quality for bone health and maintenance

Dietary protein is essential to sustain the bone protein matrix, facilitating its turnover and remodeling [52]. In addition, dietary protein also plays vital roles in the regulation of insulin-like growth factor (IGF)-1 concentrations [53], the absorption of calcium in the intestines, and the suppression of parathyroid hormone, all of which are factors that contribute to the promotion of optimal bone mineralization [[54], [55], [56]].

Interestingly, there have been various differing opinions on the effects of animal-based compared with plant-based protein foods on bone health, highlighted by Professor Kiran Bains. IGF-1 is a hormone that promotes bone mineralization, growth, and development. Some have found that animal protein, specifically meat, is associated with higher concentrations of IGF-1 [57] in contrast to soy protein [58]. Despite this, a meta-analysis of 7 different studies concluded that both animal and soy proteins do not exert significant impact on bone mineral density outcomes [59]. Although some studies suggested that an animal protein diet being worse for bone health than a plant protein diet, as there was a greater urinary calcium excretion induced by dietary animal protein [60,61], other studies found no association between reduction in bone mineral density and osteoporosis risk with higher intake of animal protein [52,62]. On the whole, animal-based and plant-based foods fulfill different roles in our diets, and both are beneficial for bone health.

#### Micronutrients availability in animal-based compared with plant-based protein foods

The discourse regarding animal-based and plant-based protein sources will also need to include plant-based meat alternatives (PBMAs) as the latter presents a potential alternative to bridge the predominantly meat-based protein consumer to plant-based diets. However, the long-term health impacts of PBMAs, in its current forms, are still unknown [63]. Furthermore, it is important to consider that many PBMAs currently available in the market go through extensive manufacturing processes, which strip them of the bioactive components (such as dietary fibers, phytochemicals, vitamins, and minerals) in their natural food matrix, crucial for the various health benefits imparted by plant-based diet [63].

A recent modeling study by Tay et al. [64] observed that a gram-to-gram substitution of animal-based ingredients in a typical 4-d diet of Singaporeans to PBMAs saw significant increases in carbohydrate, dietary fiber, and sodium; with a decreased intake in energy, protein, and fat. Unexpectedly, they also saw a significant increase in calcium intake with the substitution from animal to PBMAs, which they attributed to fortification by manufacturers or the addition of calcium as part of the manufacturing process [64]. The outcome of the modeling study contrasted with the result of a 12-wk randomized controlled trial where partial replacement of animal-based proteins to plant-based proteins increased bone resorption markers in healthy adults, attributed to lower calcium and vitamin D intake [65]. These 2 contrasting results highlight the complexity of comparing animal-based and plant-based diets. PBMAs manufacturing process is a double-edged sword: with potential for fortification as the benefit whereas the removal of various bioactive compounds and addition of sodium to be the disadvantage. Vitamins and minerals such as vitamin B-12, potassium, calcium, magnesium, and zinc were the nutrients of concern when switching from animal-based to plant-based foods because of the lower contents or bioavailabilities of particular nutrients within plant foods [66,67]. Micronutrient deficiencies remain to be a substantial public health challenge in SEA, especially in women of reproductive age and children <2 y children with food-based recommendations often involving fortification and animal-based products to meet these groups’ requirements [68]. In Singapore, the National University of Singapore Heart Study observed that plasma mean vitamin B-12 concentrations were lowest among Indians and it was attributed to the higher prevalence of vegetarianism among this population group based on food consumption surveys [69]. Fortification is especially crucial and can circumvent this issue, but fortification programs vary from country to country, needing harmonization.

#### Animal-based compared with plant-based protein foods and metabolic health

Plant-based diet has been linked to improvement in cardiovascular outcomes as well as longevity [33]. However, Tay et al. [64] highlighted that a gram-to-gram replacement of animal-based to plant-based foods significantly increased the carbohydrate intake. Carbohydrate restriction has been identified to be an effective approach in maximizing metabolic benefits associated with obesity and type 2 diabetes mellitus [70]; hence, an increase in carbohydrate from the switch of animal-based to plant-based foods may further exacerbate the high prevalence of diabetes and abdominal obesity in SEA [71]. Second, the increase in sodium reported by Tay et al. [64] may also increase cardiometabolic disease risk associated with high-blood pressure, which is already higher among Southeast Asians [71].

#### Protein foods: cost and sustainability

The argument against consumption of animal-based protein foods on environmental footprints has mostly been made using the metrics of gross protein requirements (uncorrected for digestibility and utilizability) [72]. Prof Paul Moughan highlighted the need to account for protein digestibility and utilizability when considering the impact of food production on environmental footprints. When protein quality considered, the argument shifts in favor of animal-based proteins. Comparing pork with maize production shows that the former produces less greenhouse gases per kilogram of digestible lysine (often the first limiting amino acids in humans) [72]. Similarly, eggs also produce less greenhouse gases and require much less freshwater per kilogram of digestible lysine [72]. Another data set [73], which considered the heterogenous and variable food production systems for a single food, also corroborated this finding, with eggs, farmed fish, poultry, and pork producing less carbon dioxide emissions compared with the production of soymilk [72].

Additionally, life cycle assessment (LCA) using priority micronutrient value [74] found that some animal-based protein foods’ environmental footprints are comparable with their plant-based alternatives [75]. An example is egg’s carbon footprint being 42% lower than tofu when assessed using priority micronutrient value compared with 48% higher when assessed by mass [75]. This stark difference in the outcome calls for methodological standardization when conducting LCAs and other sustainability measures. In fact, Professor Sarah McLaren stressed on the importance of using nutritional LCA as a method to assess the possible alternatives to animal-based protein diets. A composite parameter also needs to be developed to represent the complex interplay between various nutrients within a food, such as between protein and micronutrients bioavailability [76].

Henceforth, it is crucial to analyze the overall environmental impact of food production based on accurate parameters, that is, protein quality and quantity [76]. It is also important to note that each country or region has its own food production methods as well as specific nutritional needs and dietary patterns. Therefore, development of agrifood policies in individual countries or regions should not be based on global mean footprint values [75]. It is important to note that a shift toward a plant-based diet replacing proteins from animal-based sources may exacerbate the problem of protein undernutrition in developing countries who are already not consuming sufficient protein. As mentioned earlier, widespread protein inadequacy is still observed in developing countries in SEA such as the Philippines, Indonesia, and Vietnam [10,12,13]. A narrative pushing for adoption of plant-based protein diet in the name of health and sustainability in the SEA region is ignorant of the underlying issue of malnutrition and the deeper issue of the wide socioeconomic divide within the region.

Another important consideration to be made is the cost and affordability of plant-based diet (including PBMAs). Least-cost diet is defined as the cheapest diet to meet the nutritional needs of human adults. A linear programming analysis performed in the United States and New Zealand showed that animal proteins are needed for least-cost diet to be achieved [77]. Additionally, novel PBMAs’ higher cost has been continuously cited to be one of the barriers to switching [78].

## Future Food Research to Improve Protein Nutrition: Toward Functionality, Consumer Acceptance, and Safety of Complementary and Alternative Proteins in SEA

Advancements in food science and technology are key to enhancing the nutritional value of currently available food forms and the novel food sources, such as PBMAs. These can involve improving protein quantity, amino acid profiles, vitamin content, and overall digestibility to more closely mimic the nutritional benefits of animal proteins.

Despite this growing interest, PBMAs industry faces challenges in achieving broader public acceptance. Dr. Christian Carrillo gave a summary on the challenges accompanying the advancement of PBMAs, encompassing the identification of protein sources, enhancement of taste profiles, fortifications of beneficial constituents, and the optimization of nutritional compositions. Notably, plant proteins often have poor functionality, such as insolubility, limiting their use in food products. To tackle these challenges, Dr Shaun Sim presented on emerging strategies such as high-pressure processing and deep eutectic solvents which aim to boost these proteins' functionality. Concurrently, the refinement of scalable separation techniques remains instrumental in harnessing plant proteins’ full potential [79].

Another significant hurdle in PBMAs production is the necessity to incorporate fibrous textures reminiscent of meat and the use of culinary cues to neutralize plant foods’ innate flavors [80]. To address these challenges, it is important to have a better understanding of the protein sources’ unique properties. Reverse food engineering enables the characterization of different food attributes such as in sensomics, metabolomics, food–microbiome interaction, and biophysical characterization. The application of several technological processes, including extrusion, molding, and 3-dimensional printing play crucial roles in this characterization and the structural development of future foods [81]. An ongoing initiative called Proteins 4 Singapore, which used this principle, was introduced by Dr. Stefan Klade.

Sensomics, a detailed exploration of protein flavors and aromas, is pivotal in bolstering the consumer acceptance of novel protein foods. Dr Florian Utz presented an example of sensomics work to decode pea proteins’ flavor. By analyzing volatile and nonvolatile profiles of pea protein, key odorant compounds contributing to bitter off-taste were identified [82]. This offers opportunities to refine the production process to reduce the bitterness of pea protein and to enhance the overall acceptance of PBMAs.

The intersection of health and sustainability underscores consumers’ interest in plant-based diets [82]. In steering PBMAs’ development, it is crucial to prioritize nutritional integrity while mitigating overprocessing that could potentially compromise their benefits. Additional considerations including cultural acceptability, economic fairness, and regional ingredient accessibility also play significant roles in ensuring widespread adoption of PBMAs [83]. In this regard, SEA has enormous potential for various unexplored protein sources. There exists a plethora of starchy roots and leaves, and pods from underused plant-based protein sources in Indonesia, which can supplement iron, zinc, calcium, niacin, and folate when consumed as complementary protein sources [84].

In addition to PBMAs, cultivated meat is another promising source of complementary and alternative protein as it offers similar sensory profiles to the later. It also has the potential to become a more sustainable food production paradigm once the biomanufacturing processes are optimized. The biomanufacturing process encompasses cell isolation and banking, the use of edible biomaterials scaffolds for structure formation, and biofabrication approaches to create the final meat product [85]. Concurrently, the development of serum-free media and bioreactor design are essential for efficient and scalable production [85]. Although there have been advancements in small-scale cell culture optimization, large-scale bioreactor development remains a notable gap. This limitation was highlighted by Dr Deepak Choudhury to be a vital frontier for research to ensure the commercial feasibility of cultivated meat.

On the novel food aspect, proteomics is a leading approach used in the evaluation of newly discovered proteins sources from pulses, insects, or algae. Proteomics enable comprehensive analysis of proteins, providing valuable information on protein composition, sensory attributes, presence of antinutritional components, and probable allergenicity. Potential allergenic risks or cross-reactivity of black soldier fly proteins have been discovered using proteomics by characterizing these proteins [83]. Professor Michelle Colgrave explained that combining allergenicity prediction with food processing offers the opportunity to optimize food processing for potential allergens reduction or elimination. This ensures the safe consumption of novel protein such as algae-based or insect-based proteins.

Finally, it is of paramount importance that these novel complementary and alternative proteins undergo rigorous safety evaluations. Dr. Benjamin Smith highlighted the need to have a concentrated focus on potential toxins and allergens inherent in these novel products while bringing them to the market. Various assessments methods exist to evaluate the toxicity and allergenicity of individual proteins. Comparative sequence analysis to known allergens and toxins via bioinformatics (e.g., AllerCatPro) can identify similarities in the sequence, folding, and 3-dimensional structures and epitopes of the particular protein. Furthermore, it is also crucial to understand how the proteins are digested as various peptide sequences (as products of digestion) may be allergenic. These evaluations serve as a foundation for assessing risks and play instrumental roles in effectively communicating the viability and safety of these novel protein sources, with the hope of subsequently increasing the widespread adoption of these novel protein products.

## Key Recommendations and Summary

Table 1 [33,40,63,64,66,72,86–94] summarizes and compares the key differences among animal protein, plant protein, and PBMAs. Understanding the benefits and challenges of switching from animal-based to plant-based diet, it is perhaps advantageous for us to view plant-based foods and PBMAs as complements instead of total replacements to our current predominantly animal-based diet. Metabolomics comparison between grass-fed meat and PBMAs shows clear distinction (90%) between metabolites profiles despite comparable macronutrients values [95]. This finding further supports the importance of both animal and plant sources of protein and their complementary roles in sustaining both human and planetary health, a view that has also been supported by others [96].

**TABLE 1:** A comprehensive comparison of the health and sustainability implications associated with animal-based protein, plant-based protein (protein sources from whole foods), and plant-based meat alternatives.

Key indicators	Animal-based protein	Plant-based protein	PBMAs
Physiological health			
Saturated fats and cardiovascular health	High in saturated fat content, associated with cardiac mortality [33]	Absence of saturated fat leads to better lipid outcomes [33]	Varies by preparation, similar to or lower than animal-based protein [[86], [87], [88]]
Protein quality and amino acids adequacy	Complete amino acids profile benefits muscle health and growth [40]	Incomplete amino acids profile; requiring complementary protein sources [40]	A mixed plant protein that include various plant protein isolated from various sources has potential to create plant protein matrices with good protein quality, but further investigation is needed to study the antagonistic effects of antinutritional compounds on the bioavailability of plant proteins [89]
Dietary fiber	Absence of dietary fiber, increasing risk of metabolic diseases [33,90,91]	Contribute to higher fiber intake associated with various metabolic health improvement [92]	Varied depending on protein source and if fiber is added as an ingredient during processing [93]
Phytochemicals	Absence of phytochemicals’ protective effect, along with increased intake of dietary cholesterol and saturated fat associated with increased metabolic health risk [91]	Bioactive components, such as soy isoflavones contributing to better metabolic health outcomes [63,91]	Protein isolates manufacturing process strips the bioactive components [63]
Vitamins	Micronutrients vitamin B-12 and zinc are commonly found in animal foods [66]	Require supplementation [66]	Often manufactured with fortification in place [64]
Trace elements	High sodium leading to poorer health outcomes [66] High in iron and zinc as animal meats are rich in both minerals. Excessive heme iron consumption, however, has been linked to cancer and diabetes [66]	Low sodium leading to better health outcomes [66] High in nonheme iron from legumes and seeds [66] Adequate in zinc [66]	High sodium leading to poorer health outcomes [64] High in nonheme iron due to frequent inclusion of plant nonheme iron or ferrous sulfate [66] in PBMAs Inadequate in zinc [66], which may lead to poorer health outcomes such as diarrhea in infants and children; alopecia, delayed growth and frequent infections in older children; delayed wound healing in older adults [94]
Planetary health	Sustainability should be determined using amino acid instead of protein requirements. Eggs, farmed fish, poultry, and pork produce lower greenhouse gases than soymilk production [72]	More sustainable than beef production [72]	Extensive manufacturing processes may lead to lower sustainability [72]

Abbreviation: PBMA, plant-based meat alternative.

At the same time, innovation is especially needed in the manufacturing and creation of novel PBMAs that can better meet the nutritional requirements of humans while maintaining the benefits associated with plant-based foods’ bioactive compounds. Professor Christiani Jeyakumar Henry highlighted the importance of deconstruction and reconstruction of different ingredients to develop alternative protein foods; whereas Dr. Umi Fahmida showed the potential of harnessing the concept of food Multimix and identifying locally available but underused food sources to address the differing nutrient adequacies of the communities [97,98]. Gentle ingredient processing such as dry fractionation could help preserve micronutrients and fiber in the plant protein ingredients. Combining protein sources from both animal and plant to create hybrid protein foods, which encompass benefits from both, may also be beneficial in addressing the shortcomings of either source of protein.

In summary, addressing protein requirements within the Southeast Asian region should encompass both quantity and quality adequacy. Beyond that, another important factor lies in ensuring accessibility, especially in the backdrop of socioeconomic disparities. Moreover, sourcing these proteins should resonate with environmental sustainability, placing minimal strain on planetary resources. Adapting a mixed food models and exploring diversity of local (country-specific) and regional existing and novel protein sources hold potential in offering sustainable alternative protein sources.

Furthermore, as the paradigm shifts toward complementary and alternative novel protein sources, it is imperative that innovation persists to create food products that are nutritious, sustainable, and widely accepted by the public. Food science and technology should continue to be used to optimize products to address consumer acceptance. To avoid overprocessing of the food products, one should consider focusing on devising unique and enticing food categories, rather than merely emulating traditional meat products.

Finally, food safety concerns are vital in ensuring the successful adoption of plant-based food alternatives. Although alternative proteins present promising solutions to sustainability and health issues, there are still many challenges to be tackled. Meeting these challenges will necessitate a synergistic endeavor involving innovators, regulatory bodies, and educators to guarantee that these emerging food sources are not only safe and nutritious but also palatable to consumers and conducive to enhance the robustness of our global food systems.

## Author contributions

The authors’ responsibilities were as follows – AST, JYW, SH: conceptualized research; AST, JYW: wrote the original and final draft; GS, CA, ABM, SYJS, RT, SH, CJH: reviewed and edited the paper; and all authors: have read and approved the final manuscript.

## Conflicts of interest

The authors report no conflicts of interest.

## Funding

This research was supported by the Agency for Science, Technology and Research (A∗STAR), under the Singapore Food Story R&D Programme, SFS-2 IAF-PP Future Foods: Alternative Proteins (H20H8a002). The symposium and publication fee were funded by ILSI which is supported by its industry members.
